# Chemical vs entomopathogenic control of *Thaumastocoris peregrinus* (Hemiptera: Thaumastocoridae) via aerial application in eucalyptus plantations

**DOI:** 10.1038/s41598-019-45802-y

**Published:** 2019-07-01

**Authors:** Carlos Frederico Wilcken, Mário Henrique Ferreira do Amaral Dal Pogetto, Alexandre Coutinho Vianna Lima, Everton Pires Soliman, Bianca Vique Fernandes, Isabel Moreira da Silva, Antonio José Vinha Zanuncio, Leonardo Rodrigues Barbosa, José Cola Zanuncio

**Affiliations:** 1 Departamento de Proteção Vegetal, FCA/UNESP, Universidade Estadual Paulista, Botucatu, São Paulo Brazil; 2 Dow Agrosciences, Mogi-Mirim Field Station, Mogi-Mirim, São Paulo Brazil; 3 MIP Florestal, Ubirajara, São Paulo Brazil; 4 JB Biotecnologia, Paraopeba, Minas Gerais Brazil; 5 Departamento de Fitotecnia, Universidade Federal de Viçosa, 36570-900 Viçosa, Minas Gerais Brazil; 6 Departamento de Engenharia Florestal, Universidade Federal de Viçosa, 36570-900 Viçosa, Minas Gerais Brazil; 7 Empresa Brasileira de Pesquisa Agropecuária- Embrapa Florestas, 83411-000 Colombo, Paraná Brazil; 8 Departamento de Entomologia/BIOAGRO, Universidade Federal de Viçosa, 36570-900 Viçosa, Minas Gerais Brazil

**Keywords:** Animal behaviour, Entomology, Animal behaviour, Entomology

## Abstract

The *Thaumastocoris peregrinus* spread to eucalyptus plantations in many countries. Chemical control is a questionable measure, mainly due to the environmental impact, high cost and moreover has the use restricted by the forest certifications. Bio-insecticides may have similar efficiency to chemical products to control *T. peregrinus*. The chemical thiamethoxam, thiamethoxam + lambda-cyhalothrin, acephate and the microbial *Beauveria bassiana* and *Metarhizium anisopliae* insecticides were tested at different doses to manage *T. peregrinus*. The products were sprayed on eucalyptus plants using aircraft and populations of this insect were counted before application and at 1, 14 and 21 days afterwards (DAA). Ten eucalyptus trees were evaluated per plot, with the collection of ten leaves from the middle third of the crown of each tree, and the number of *T. peregrinus* nymphs and adults obtained per leaf was determined. All the chemical insecticides had similar control at 1 DAA for *T. peregrinus* nymphs and adults. At 14 DAA, the number of *T. peregrinus* nymphs and adults on eucalyptus leaves was similar for the chemical and microbial insecticide treatments. At 21 DAA the control efficiency of *T. peregrinus* nymphs and adults was higher than 80% with all insecticides. The entomopathogenic insecticides have potential for aerial application to control *T. peregrinus* nymphs and adults and provide viable and environmentally-friendly alternative to manage this pest.

## Introduction

*Eucalyptus* (Myrtales: Myrtaceae), a native plant mainly from Australia, was introduced to Brazil and planted in genetically homogeneous and continuous areas to produce raw material for the forestry industry^1,2^. Eucalyptus plantations occupy 5.7 million hectares, representing 72% of the total planted trees in this country^3^. Homogeneous forests may be more susceptible to pests^4–6^ reducing productivity of Eucalyptus plants^7–9^.

*Thaumastocoris peregrinus* Carpintero & Dellapé 2006 (Hemiptera: Thaumastocoridae) is a serious pest with a rapid dispersal rate in eucalyptus species and hybrids due to high reproductive capacity, rapid colonization and broad infestation^10,11^. An Australian native, this pest spread to South Africa, Zimbabwe, Malawi, Kenya^12^ and to countries such as Argentina^13^, Brazil^14,15^ Chile^16^, Italy^17^, New Zealand^18^, Portugal^19^, Uruguay^20^ and Mexico^21^. In Brazil, this insect was detected in 2008 in Rio Grande do Sul and São Paulo states and later in Minas Gerais, Espírito Santo, Rio de Janeiro, Mato Grosso do Sul^15^, Goiás^22^, Paraná^14^, Santa Catarina^23^ and Sergipe^24^ states.

The short life cycle and high reproductive potential facilitate the rapid population growth of *T. peregrinus* in the field^10,11,25,26^, reducing photosynthetic apparatus an thus tree growth^15^ and productivity^27^. The analysis of the ecophysiological variables allows evaluating damages to the photosynthetic ability of *E. camaldulensis* by the bronze bug attack^27^. Sap sucking by *T. peregrinus* nymphs and adults causes chlorotic spots, leaf fall and decreases photosynthetic area^12,14,17^, which can lead to plant death^15^. Leaves damaged by *T. peregrinus* are initially silver, subsequently turning brown and red, which gives the tree a bronzed appearance, justifying its common name as bronze bug^15^. *Eucalyptus* species planted in Brazil includes *Eucalyptus camaldulensis*, *Eucalyputus grandis* and *Eucalyptus urophylla* and hybrids adequate for the *T. peregrinus* development and reproduction^26^.

Chemical control is, usually, used in insect population outbreaks and *T. peregrinus* was managed in urban areas with the systemic imidacloprid insecticide^28^. In Brazil the pyrethroid Capture 400 EC (FMC Agricultural Solutions) is the only product registered to control *T. peregrinus*. However, chemical control can cause environmental impact including reduction of natural enemies, intoxication of users and environmental contamination by the use of these products in extensive areas and moreover they have high cost and are restricted by the forest certification bodies^29–31^. Aerial spraying with insecticides may impact wildlife and beneficial insects^32^. The issue with aerial applications of neonicotinoids is related to its drift, gradual accumulation in target crop and non-crop vegetation, phloem-mediated translocation to nectar or pollen, the subsequent lethal and sub-lethal impacts on herbivores and higher trophic levels (including birds and arthropod natural enemies)^32,33^. Thus it is necessary to propose alternative control which are efficient, cost-effective and environmentally sound^34,35^.

Efficient strategies to manage the *T. peregrinus* in commercial plantations in Brazil are unavailable, thus, biological control is the viable option against this pest^30,36^. The *Cleruchoides noackae* Lin and Huber (Hymenoptera: Mymaridae)^12^, *Hemerobius bolivari* Banks (Neuroptera: Hemerobiidae)^19^, *Chrysoperla externa* (Hagen) (Neuroptera: Chrysopidae)^15^ and predatory stinkbug have been reported as natural enemies of *T. peregrinus*^19,37,38^. In Brazil, microbial control is a viable alternative due to favorable environmental conditions. Entomopathogenic fungi are used against agricultural insect pests, because they are natural to the environment. *Beauveria bassiana* (Bals.) Vuillemin and *Metarhyzium anisopliae* (Metsch.) Sorokin have wide host range^39^. They are used via inoculative, conservative, incremental or inundative applications and penetrate host integument^40^. *Metarhizium anisopliae* and *B. bassiana* are effective against forest pests^41–43^. However, it is important to determine the concentrations of mycoinsecticide to overcome natural host defense mechanism barriers and to cause host death^40,44^. The importance of the forestry sector to the Brazilian economy and the introduction of *T. peregrinus* into Brazil make it necessary to reduce population outbreaks of this pest.

The objective of this study was to investigate the efficiency of entomopathogenic fungi compared to chemical insecticides to control *T. peregrinus*.

## Results

The number of *T. peregrinus* nymphs and adults per eucalyptus leaf, before application, was similar between treatments (Table 1).

**Table 1:** Mean number of *Thaumastocoris peregrinus* (Hemiptera: Thaumastocoridae) nymphs and adults per eucalyptus leaf in the biological and chemical insecticide treatments before application and at 1, 14 and 21 day after application.

Treatment	Before	1 DAA^a^	14 DAA	21 DAA
*Beauveria* 0.5 Kg/ha	7.52 a	—^b^	0.30 ab	0.17 a
*Beauveria* 1.0 Kg/ha	8.03 a	—	0.26 a	0.44 ab
*Beauveria* 1.5 Kg/ha	5.28 a	—	0.65 ab	1.59 cd
*Metarhizium* 0.25 Kg/ha	0.17 a	—	2.08 b	1.27 bcd
*Metarhizium* 0.50 Kg/ha	0.44 a	—	0.30 ab	1.54 cd
*Metarhizium* 1 Kg/ha	1.59 a	—	0.63 ab	2.09 d
Actara (thiamethoxam) 0.1 Kg/ha	5.91 a	0.69 a	0.27 a	1.42 cd
Actara (thiamethoxam) 0.15 Kg/ha	3.54 a	0.74 a	2.00 ab	0.93 abc
Actara (thiamethoxam) 0.2 Kg/ha	5.73 a	0.75 a	0.94 ab	1.56 cd
Engeo Pleno (lamb. + thiam.) 0.2 L/ha	6.06 a	0.32 a	0.60 ab	0.43 ab
Orthene (acephate) 0.5 Kg/ha	2.96 a	0.90 a	0.58 ab	0.16 a
Control	2.61 a	2.95 b	1.36 ab	4.14 e
CV (%)	40.01	17.68	23.32	12.09
*F*	0.61^ns^	9.90*	3.28	23.45*

lamb. + thiam. = Lambda-cyhalothrin + thiamethoxam. The data were transformed (x + 0.5)^1/2^ before the statistical analysis. Means followed by the same letter per column did not differ from each other by Tukey test (p < 0.05). ^a^DAA = days after insecticide applications. ^b^Unsatisfactory evaluation in the treatments with mycoinsecticides due to insufficient time to cause insect death.

**Figure 1:**
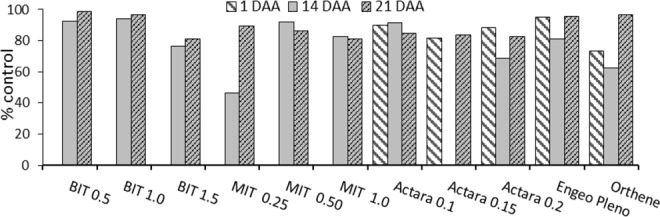
Efficiency (%) of the insecticides Boveril (BIT 0.5 = BIT 0.5 kg/ha, BIT 1.0 = BIT 1.0 kg/ha, BIT 1.5 = BIT 1.5 kg/ha), Metarril (MIT 0.25 = MIT 0.25 kg/ha, MIT 0.50 = MIT 0.50 kg/ha, MIT 1.0 = MIT 1.0 kg/ha), Actara 0.1 = Actara (thiamethoxam 0.1 kg/ha), Actara 0.15 = Actara (thiamethoxam) 0.15 kg/ha, Actara 0.2 = Actara (thiamethoxam) 0.2 kg/ha, Engeo Pleno = Engeo Pleno (lambda cialothrin + thiamethoxam) 0.2 l/ha e Orthene = Orthene (acephate) 0.5 kg/ha to control *Thaumastocoris peregrinus* (Hemiptera: Thaumastocoridae) nymphs and adults in the first (1 day after application = DAA), second (14 DAA) and third (21 DAA) evaluations (Henderson-Tilton formula).

The number of *T. peregrinus* nymphs and adults per leaf was lower 1 day after the application of the insecticides thiamethoxam (Actara), thiamethoxam + lambda cyhalothrin (Engeo Pleno) and acephate (Orthene) (Table 1), observing greater efficiency with the second product (Fig. 1). The control efficiency was 73; 81; 88; 90 and 95% for the acephate (Orthene), thiamethoxam (Actara) and Engeo Pleno at the rates of 0.15, 0.2 and 0.1 kg/ha, respectively (Fig. 1).

Fourteen days after the application of the chemical and biological insecticides, the number of *T. peregrinus* nymphs and adults were similar (Table 1). The efficiency of thiamethoxam + lambda-cyhalothrin (0.2 L/ha), *M. anisopliae* (1 kg/ha), thiamethoxam (0.1 kg/ha) and *B. bassiana* (0.5 and 1 Kg/ha) was 81, 83, 91, 92 and 94%, respectively (Fig. 1).

The number of *T. peregrinus* nymphs and adults at 21 days after application was lower for *B. bassiana* with the lowest doses and similar to the treatments with Engeo Pleno, Orthene and Actara (0.15 Kg/ha) (Table 1). The control was above 80% in all treatments, with greater efficiency for *B. bassiana* 0.5 kg/ha (99%) and acephate 0.5 kg/ha (97%) (Fig. 1).

## Discussion

Biological insecticides, such as entomopathogenic fungi, are safer and have lower health risks than chemicals in pest control^45,46^. The bronze bug mortality by *B. bassiana* and *M. anisopliae* in the field is poorly studied but microbial products may have high efficiency in the integrated management of this pest in forest crops. Temperatures of 27, 28 and 29 °C and precipitation of 20; 25 and 63 mm in August, September and October were adequate for sporulation and favored the control efficiency of *T. peregrinus* by *B. bassiana* and *M. anisopliae*, as observed in other works^46,47^. These entomopathogens have a wide host range, but their germination, conidia persistence, host mortality and sporulation depend on adequate environmental conditions such as temperature and humidity^48,49^. *Beauvaria bassiana* and *M. anisopliae* can grow between 5 to 30 and 5 to 40 °C respectively, but showing optimal growth at temperatures of 25 and 30 °C^50^, determining their efficacy in biological control. This makes necessary an adequacy between the temperature and humidity for the efficiency of these fungi. Entomopathogen applications in the field are preferable in the late afternoon to avoid the negative impact of abrupt changes in temperature^51^. The low precipitation level may initially compromise the spore penetration and survival even at adequate temperatures. However, increased precipitation favored conidiogenesis in the first dead individuals, with horizontal transmission and dissemination of the disease throughout the populations. This is important because the potential for fungus conidiogenesis is determinant in the pathogen spread among pest individuals.

The higher efficiency of the chemical insecticides, 1 day after application, shows its faster impact due to the pyrethroid and neonicotinoid knock-down effect. This is similar to other neonicotinoids and pyrethroids such as imidacloprid and lambda-cyhalothrin against *Bucephalogonia xanthophis* (Berg 1879) (Hemiptera: Cicadellidae) with mortality above 90% in 24 h and near 100% within 48 h^52^. The neonicotinoid-pyrethroid mixture (thiamethoxam + lambda-cyhalothrin) increased the control efficiency of *T. peregrinus* with results similar to that observed for *Myzus persicae nicotianae* Blackman 1987 (Hemiptera: Aphididae) up to 70 DAA in tobacco crop^53^. Imidacloprid and thiamethoxam are systemic insecticides of the neonicotinoid group that act as an acetylcholine agonist in the synapses of the central nervous system^54^. The pyrethroid lambda-cyhalothrin is a sodium channel modulator, causing repetitive and uncontrolled impulses, hyperexcitation and death^55^. However, these insecticides have rapid action and often are toxic to beneficial organisms. Bees may come into direct contact during pollen and nectar collection or through contaminated water^32,33^. Thiamethoxam is toxic to parasitoids and predators in forest environments and agricultural crops^56^. In addition, airborne application with chemical insecticides may aggravate the situation by contaminating nearby wild and cultivated plants^33^.

The similar control efficiency of *B. bassiana* and *M. anisopliae* entomopathogens and chemicals for the *T. peregrinus* nymphs and adults after 14 DAA suggests efficiency of the microbial control. The horizontal transmission and dissemination of diseases in pest populations determine the pathogen efficiency being favored by greater humidities^57^. Delayed effects via horizontal transmission for entomopathogens have been reported for *M. anisopliae* on *Oncometopia facialis* (Signoret) (Hemiptera: Cicadellidae)^58^ and *B. bassiana* in *Bemisia tabaci* (Gennadius, 1889) (Hemiptera: Aleyrodidae)^45^. Epizootic occurrences of Entomophtorales fungi were reported for *T. peregrinus* nymphs and adults in a *Eucalyptus* plantation in São Paulo state, Brazil^44^. Entomopathogen use in integrated pest management is a viable, low-risk technique and has the capacity to exploit a wide host range through different action modes^46^. Fungi cause death by penetrating and destroying the external arthropod cuticle^59,60^. The fungi *B. bassiana* and *M. anisopliae* have rapid dispersion in the field with potential to control forest insect outbreaks^40,41^, and sucking insects such as *Nilaparvata lugens* Stål (Hemiptera: Delphacidae)^61^ and *Diaphorina citri* Kuwayama (Hemiptera: Liviidae)^62^. The gregarious behavior of *T. peregrinus*^25,28,63^ may facilitate entomopathogenic fungal epizootics in the field. The entomopathogenic fungi such as *M. anisopliae* and *B. bassiana* are effective against pest insects but they can affect natural enemies. This suggests a careful criterion in using these fungi to maintain the effectiveness of the control exerted by both.

The control efficiency of *T. peregrinus*, at 21 days, with the entomopathogen products, mainly for *B. bassiana*, shows residual effects and the horizontal dispersion capacity of this fungus as reported against *Bucephalogonia xanthophis* (Berg) (Hemiptera: Cicadellidae)^45^. Entomopathogenic fungi are slower acting and need higher relative humidity and/or rainy periods. Additionally, they require longer periods to cause mortality compared to synthetic chemical products^64^, but side-effects in infected insects reduces feeding and damage^65^. The pathogenicity and virulence of the mycoinsecticides indicate that the fungi overcome the physical barriers such as insect sclerotic exoskeleton as found for the natural occurrence of a fungus from the order Entomophthtorales on *T. peregrinus* in São Paulo state, Brazil^44^. The tegument may act as a physical barrier to the penetration and the germinative tube or may have chemical properties inhibiting conidia germination. *Thaumastocoris peregrinus* control over 80% at 21 DAA using entomopathogenic fungi indicates the delayed effect of this product as found for *B. bassiana* surviving and colonizing foliar tissues 30 days after inoculation without damaging plants^66^.

*Beauveria bassiana* and *M. anisopliae* have with potential to control *T. peregrinus* as found against Hemiptera pests such as aphid^67^, *Riptortus pedestris* (Fabricius, 1775)^68^, *Diaphorina citri* Kuwayama^69^, *Bemisia tabaci*^70^. Certified forest companies seek practices that conserve the environment, such as integrated pest management, giving preference to biological, cultural control and the use of less toxic products. Biological control is the only viable option to manage *T. peregrinus* in commercial eucalyptus plantations reducing the toxicity drift caused by the pyrethroid and the neocotinoid in aerial applications. The compatibility of chemicals with microbial agents and the effect of these products on natural enemies need better studies for integrated pest management.

The *T. peregrinus* control was similar with entomopathogens and chemical insecticides. The efficiency of the fungi *B. bassiana* and *M. anisopliae* at lower concentrations and its high residual period shows the potential of these products to control *T. peregrinus* nymphs and adults in eucalyptus plantations with low impact on other organisms such as parasitoids and predators. The adoption of control measures may be part of integrated management programs, where other control strategies can be used in a joint manner.

## Methods

### Obtaining fungal spores

The fungus *Beauveria bassiana* (isolated ESALQ PL63- obtained from *Atta* spp. in Piracicaba, São Paulo, Brazil) was the active ingredient of the product Boveril and *Metarhizium anisopliae* (ESALQ E9 isolate - obtained from *Mahanarva posticata* in Boca da Mata, Alagoas, Brazil) that of the product Metarril. Both are deposited in the Bank of the Laboratory of Pathology and Microbial Control of Insects of ESALQ/USP Piracicaba, São Paulo, Brazil. These microorganisms were cultured by solid fermentation in rice and their conidia were dried and extracted for the assays. Spore production followed a methodology described^71^, with modifications. This methodology includes pre-baking the rice, packing it in polypropylene bags, closing the bags and sterilizing them for 20 minutes in an autoclave at 121 °C. After cooling the rice, the substrate is inoculated with microorganism strains, homogenized by manual shaking and stored in air-conditioned rooms with a controlled temperature of 25.5 ± 1.0 °C and 12 hour photoperiod and placed on shelves for four days. After this time, the rice with mycelium was spread in trays for another eight days until the conidia sporulation. After this process, the solid fermentation product is dried for three days under the same conditions of controlled temperature and photoperiod and sieved to extract the pure conidia (Personal communication, Luciano Koppert).

The pure spores of the entomopathogenic fungi were used in the same proportion of the active ingredient used in the commercial product Boveril and Metarril corresponding to 2.5 × 10^9^ spores/ha and 6.9 × 10^9^ spores/ha respectively.

### Conducting the experiment

The experiment was carried out in Pompéu, Minas Gerais state, Brazil in areas of the Vallourec & Mannesmann Florestal (V&M) with a randomized complete block design. The 12 treatments were conducted with chemical and biological insecticides with different concentrations (Table 2) with four replications and 48 plots, each 40 m wide and 500 m long, equivalent to 2 ha. The evaluation was done in the central area (2 ha) of each plot to avoid drift contamination between treatments. The clones VM01 (hybrids of *E. urophylla* and *E. camaldulensis*) with approximately 12 to 16 months old and spaced 2 × 3 m were used.

**Table 2:** Commercial products (Products), active ingredients (Ingredients), dose, date, hours of application (Hours), relative humidity (RH%), temperature in °C (Temp.) and wind speed (Wind) during aerial spraying to control *Thaumastocoris peregrinus* (Hemiptera: Thaumastocoridae) in the field. Volume of water + vegetable oil = 20 L/ha (Added 1 L/ha of vegetable oil).

Products	Ingredients	Dose	Date	Hours	R.H	Temp.	Wind
*Beauveria* ^a^	*Beauveria bassiana*	0.5 Kg/ha	09/09	16:40	48.0	28.0	4.44 m/s
		1.0 Kg/ha	09/09	17:05	50.0	27.0	4.44 m/s
		1.5 Kg/ha	09/09	17:22	53.0	26.0	4.44 m/s
*Metarhizium* ^a^	*Metarhizium anisopliae*	0.25 Kg/ha	09/09	17:40	56.0	25.0	0.55 m/s
		0.50 Kg/ha	10/09	06:48	88.0	16.0	0.55 m/s
		1.0 Kg/ha	10/09	08:03	78.0	20.0	0.55 m/s
Actara	Thiamethoxam	100 g/ha	10/09	09:36	62.0	24.0	17.22 m/s
		150 g/ha	10/09	13:00	36.0	29.0	17.22 m/s
		200 g/ha	10/09	13:18	33.0	30.0	17.22 m/s
Engeo Pleno	Thiam. + Lamb	0.20 L/ha	10/09	13:32	31.0	31.0	6.9 m/s
Orthene	Acephate	500 g/ha	10/09	13:52	30.0	31.0	5.8 m/s
Control	Water	—	—	—	—	—	—

^a^Pure spores, not the commercial product. Thiam. + Lamb. = Thiamethoxam + lambda cyhalothrin.

The products and water (control) were sprayed using an agricultural aircraft model Ipanema with Micronair AU 5000 rotary spray nozzles with electronic beacon DGPS in a round-trip evolution system with a diameter of 200 micrometer drops. After each spraying with the respective treatments, the tank was cleaned with 100 liters of water, with the aid of a tank kite. The temperature and humidity conditions in the field (Table 2) were adequate for the *B. bassiana* and *M. anisopliae* survival and development.

*Thaumastocoris peregrinus* nymphs and adults were collected before and at 1, 14 and 21 days after spraying. Microbial insecticides were not evaluated 1 day after application due to their slower action.

Ten trees were evaluated per plot with the collection of ten leaves from the middle third of the crown of each one^43^. The leaves were removed from the plant and packed in sealed paper bags which were transported to the FCA/UNESP Biological Control of Forest Pests Laboratory in Botucatu, São Paulo state, Brazil, where live insects were counted.

The mean numbers of *T. peregrinus* nymphs and adults per eucalyptus leaf were submitted to variance analysis and compared using Tukey test (p < 0.05). The control efficiency of the products was corrected by the Henderson-Tilton’s formula^72^, adequate to evaluate the number of live insects in a non-uniform population: efficiency (%) = [(numbers in the control before application x numbers in the treatment after application)/(number in the control after application x numbers in the treatment before application)] × 100}.
